# Recent Advances in Stimuli-Responsive Release Function Drug Delivery Systems for Tumor Treatment

**DOI:** 10.3390/molecules21121715

**Published:** 2016-12-20

**Authors:** Chendi Ding, Ling Tong, Jing Feng, Jiajun Fu

**Affiliations:** School of Chemical Engineering, Nanjing University of Science and Technology, Nanjing 210094, China; salamicky@sina.cn (C.D.); njustlingtong321@sina.com (L.T.); fjnjust@163.com (J.F.)

**Keywords:** drug delivery system, stimuli response, nanotechnology, anti-tumor treatment

## Abstract

Benefiting from the development of nanotechnology, drug delivery systems (DDSs) with stimuli-responsive controlled release function show great potential in clinical anti-tumor applications. By using a DDS, the harsh side effects of traditional anti-cancer drug treatments and damage to normal tissues and organs can be avoided to the greatest extent. An ideal DDS must firstly meet bio-safety standards and secondarily the efficiency-related demands of a large drug payload and controlled release function. This review highlights recent research progress on DDSs with stimuli-responsive characteristics. The first section briefly reviews the nanoscale scaffolds of DDSs, including mesoporous nanoparticles, polymers, metal-organic frameworks (MOFs), quantum dots (QDs) and carbon nanotubes (CNTs). The second section presents the main types of stimuli-responsive mechanisms and classifies these into two categories: intrinsic (pH, redox state, biomolecules) and extrinsic (temperature, light irradiation, magnetic field and ultrasound) ones. Clinical applications of DDS, future challenges and perspectives are also mentioned.

## 1. Introduction

Chemotherapy, through more than a century of development, has become a mainstream cancer therapy [1,2,3]. Benefiting from achievements in the development of anti-tumor drugs, a lot of chemotherapy regimens have been devised, showing satisfactory effects in stopping or slowing the growth of cancer cells. For example, doxorubicin (DOX) has been proved effective for breast cancer treatment [4], and 5-fluorouracil for colorectal cancer [5], epirubicin for stomach cancer [6], and paclitaxel for lung cancer [7]. However, by directly introducing these drugs into the blood stream, they can also harm healthy cells and cause severe side effects such as infections, anemia, bleeding, alopecia and nausea [8,9,10,11]. Therefore, in practical treatment, a chemotherapy course is often followed with a withdrawal period to help patients recover from the damage caused to normal cells. This, in turn, would result in a high-risk of illness relapse. The problem of rapid metabolism also undermines drug efficiency as well. All these factors have prompted researchers to seek solutions for precisely delivering anti-tumor drugs to the targeted lesion location.

Recent advances in drug delivery research bring hope for the elimination of these side-effects and maximization of efficacy. An ideal drug delivery system (DDS) should meet requirements of bio-compatibility, large drug payload and controlled release function at the same time. The carrier material itself should be chemically and biologically inert so that it would not react with the anti-tumor drugs or cause damage to human organs. The payload capacity is a key element for a successful DDS, because the dosing amount should be controlled precisely. Either too little or too much would be detrimental for the therapeutic process. To address the inefficiency issues caused by insufficient doses, materials with large drug storage volume are preferred. To avoid the risk of releasing excess anti-tumor drug, the stimuli-responsive controlled release function is imperative. By answering the stimuli signals existing in the tumor environment, the drug carrier platform releases the drug to fulfill its drug delivery purpose. More importantly, zero release until stimuli signals are applied is another indispensable feature in these systems.

In recent years, many efforts have been made in the nanotechnology field, shedding new light on the creation of multifunctional drug carrier nanomaterials. These nanoscale skeleton materials bring infinite possibilities in the design of drug delivery systems. Among them, mesoporous materials [12,13,14,15,16,17,18], metal-organic frameworks [19,20,21,22], quantum dots [23], and polymers [24,25,26,27,28,29] have been successfully used as “storage pools” to accommodate drug molecules. In this review, we will firstly focus on analyzing the features of these materials such as size, cell permeability and loading volume. Their working mechanism will also be summarized: how they store the drug cargos, either by adsorption or by binding.

**Figure 1:**
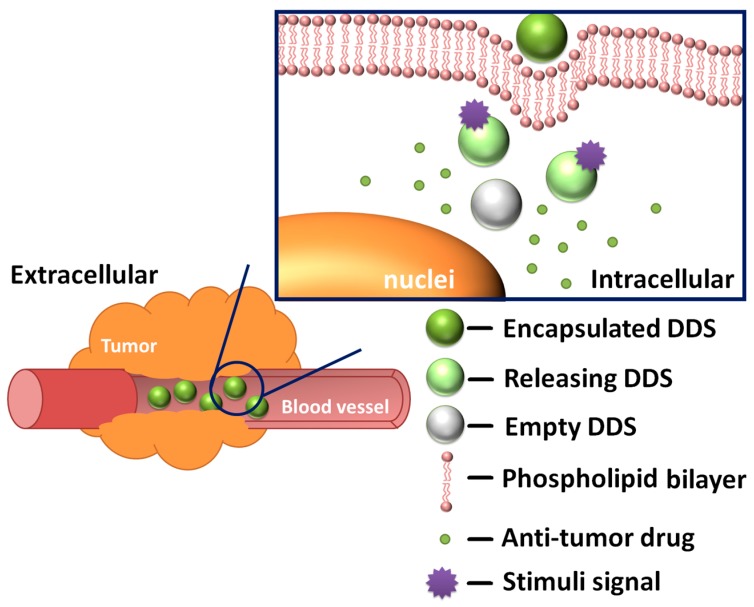
General working mechanism of drug delivery systems (DDSs).

Another vital part of a DDS is a stimuli-responsive mechanism, which determines whether the DDS is endowed with controlled release functions (Figure 1). The accurate design of a controlled release behavior should be based on a full understanding of the relevant stimuli signals and release mechanisms. DDSs that respond to pH [30,31,32,33,34], temperature [35,36,37,38,39], light [26,40,41,42,43], redox state [44,45,46,47,48,49], magnetic field [50], biomolecules [51,52,53,54] or a combination of them have already been developed and confirmed. These stimuli signals are commonly categorized into two main types: internal stimuli and external stimuli. The tremendous intracellular environment differences between the tumor tissues and the normal tissues cause heterogeneities in pH value, redox state, types and amounts of biomolecules. These natural gradients make internal stimuli an ideal trigger for controlled release and enhanced specificity against tumor cells. On the other hand, external stimuli play an equally important part—by applying extra stimuli at the disease location, scientists can have more accurate control over the DDS.

The main release modes include: gate mode, self-destruction mode and stage separation mode. In mesoporous materials, pores are used for accommodating the anti-tumor drug and “gates” are essential for regulating the flow of the drug molecules. Currently, the gate mode is the most frequently used strategy. Basically, the gates are movable entities. Whatever the form is, molecular or supramolecular switches or chemically modified nanoparticles, they should block the orifice at first then move from their original position to provide room for releasing the drug load. The self-destruction mode is inspired by the concept of metabolism: the support materials would degrade and drug would be released once the DDS reaches the disease location. In the stage separation mode, drug molecules are attached to support materials by stimuli-responsive, rupturable bonds. The release process is much like a rocket launch, leading to the detachment of the anti-tumor active parts.

Studies on stimuli-responsive DDS have made great significant progress in recent years [55,56,57,58]. Both in vitro and in vivo experiment results show its promising prospects [59,60,61]. However, researchers are still facing many challenges, for example, the precise control of release behavior and location targeting [62]. The main focus of this review is to classify and analyze DDSs structurally and functionally, presenting recent achievements on each major category. The main challenges are summarized as well.

## 2. Major DDS Scaffolds

A nanostructured scaffold is the most important constitutional part of a DDS. According to recent studies, a suitable scaffold could bring benefits such as enhanced permeation and retention effect (EPR) [63], tumor specificity [64], tunable pharmacokinetics [65] and eventually alleviation of chemotherapy side effects. There are several evaluation parameters that are crucial to the evaluation of a DDS: size, loading capacity, and loading mode. In terms of size requirement, the Royal Society and Royal Academy of Engineering gave the definition that the size of nanoparticles used for medical purpose should generally be lower than 100 nm [66]. Theoretically speaking, a smaller size means a higher surface to mass ratio and greater potential for carrying more drug molecules. Besides, researches showed that a smaller scale is also conducive to stronger penetration ability [67]. Apart from the size, the loading capacity is another vital parameter that directly measures the ability of carrying cargo molecules. There are two types of loading mode: adsorption and bonding [68,69]. The most used strategy is adsorption because of the simplicity in its implementation process and its capability of carrying large amounts of cargo molecules. However the physical adsorption interaction is not specific enough to prevent undesirable leakage. The bonding mode is not affected by this restriction, but this mode imposes strict requirements on the structure of drug molecules: the bonding site must not affect the antitumor activity site inside a molecule. Also, this mode is affected by the limitations of bonding sites. Some representative drug delivery systems are shown in Table 1. In this section, we will summarize recent research trends and give an introduction to the general working mechanism of each type of nanoscale scaffold.

### 2.1. Mesoporous Nanoparticles

The term mesoporous structure refers to pores with diameters between 2 and 50 nm [70]. Inside a DDS, the pores are used for accommodating the drug molecules. For medical purposes, the applied mesoporous structure-based materials usually have pore diameters smaller than 10 nm, otherwise, the orifice would be too large to effectively encapsulate a drug, leading to undesirable pre-leakage during the translocation process. Mesoporous materials can be made up of different materials: silicon oxide [71], zirconium oxide [72], titanium dioxide [73], niobium oxide [74], aluminum-based spinel [75] and carbon [76]. Among these materials, silicon oxide has been recognized by the U.S. Food and Drug Administration (USFDA) as a safe food additive for the basic medical care [77]. It is generally accepted that silica-based materials have stability and biocompatibility advantages [78], therefore, mesoporous silica nanoparticles (MSNs) have been the most extensively studied. Besides, the various forms of MSNs further expand the potential of practical application: a hollow mesoporous structure increases the loading capacity and core-shell [79] or rattle-type [80] composite mesoporous materials have introduced new properties such as magnetism [81] and up-conversion [82].

**Figure 2:**
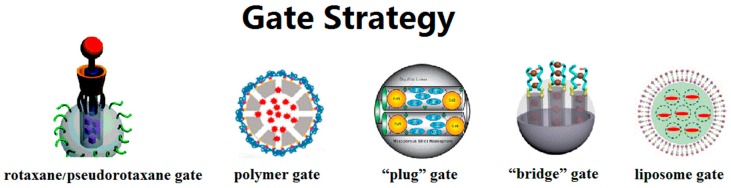
Schematic diagram of different types of gate strategies. Adapted with permission from [83,86,87,88,89]. Copyright 2010, 2015, 2003, 2015, 2016 American Chemical Society.

The gate strategy, which controls the flow of drug molecules, is the major highlight of MSN-based DDSs. The switch gates can exist in many forms: rotaxane/pseudorotaxane gates, polymer gates, “plug” gates, “bridge” gates, liposome gates, etc. (Figure 2). The switch function of rotaxane/pseudorotaxane type gates relies on the conformation change of these supramolecular assemblies. Non-covalent, intermolecular interactions such as electrostatic interaction, hydrophobic force and C–H···π interactions contribute to build the assemblies [83]. When the system is exposed to external stimuli, the original intermolecular forces are interrupted, resulting in conformational changes of the rotaxane/pseudorotaxane. On the orifice of MSNs, this changing conformation can change the blocking effect toward the internal drug molecules. Our research group has developed several such gates on MSNs. In 2014, we developed pseudo[1]rotaxane supramolecular nanovalves [84]. Anchoring a β-cyclodextrin derivative, mono-2-*O*-{1-(1*H*-benzoimidazol-2-yl-methyl)-1*H*-[1,2,3]triazole-4-ylmethyl}-β-cyclodextrin (BZI-βCD) onto the outside surface of MSNs through covalent bonds, the cavities of BZI-βCD were connected with the mesopores of MSNs. Under neutral conditions, the benzimidazole part would embed into the cavities of BZI-βCD driven by hydrophobic forces. When placed in an acidic solution, the protonated benzimidazole part detached from the cavity of BZI-βCD due to the decreased affinity constant. Therefore, the acid stimulus removes the blocking benzimidazole part and releases the *p*-coumaric acid molecules inside through the cavities of BZI-βCD. In our recent research, we installed another pseudo[2]rotaxane supramolecular nanovalve [85]. The release behavior was also triggered by a pH-caused conformation change: we realized switch binding between the novel macrocyclic molecule-carboxylic water-soluble pillar[5]arene (WP5) and the stalk molecule 1,6-(1-pyridiniumbromidehexyl)-hexanediamine (APy-HDA) with two different sites. In vitro experiment results show that DDSs with this gate have ablation ability towards MCF7 cells.

**Figure 3:**
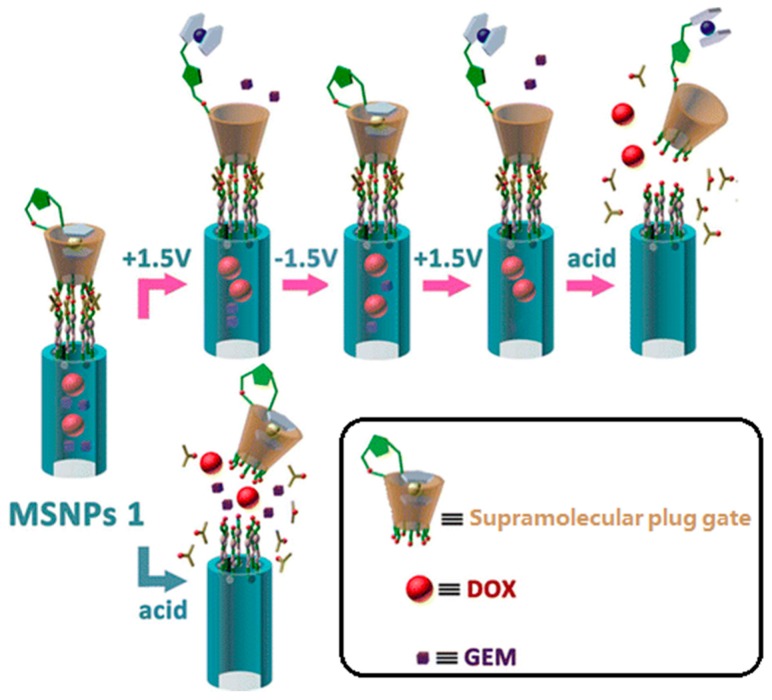
Schematic diagram of a macrocyclic molecule derivative plug gate. DOX, doxorubicin; GEM, gemcitabine. Reprinted with permission from [94]. Copyright 2015 American Chemical Society.

We define those strategies which adopt materials with the same size as mesopores (ca. several nanometers) for blocking as “plug” gates. Several kinds of “plug” have been proved viable in controlled release. At an early stage, Lin and coworkers prepared mercaptoacetic acid-coated CdS nanocrystals as plugs for preventing premature drug release [87]. Test results showed that this material has an average diameter of 2.0 nm, which is close to the average pore diameter (2.3 nm). These plugs could be removed by disulfide reducing agents. This design idea inspired many subsequent studies. Up to now, superparamagnetic Fe_3_O_4_ nanoparticles, quantum dots, dendrimers, gold nanoparticles and macrocyclic molecule derivatives have been successfully introduced onto the outer surface of MSNs to function as plug type gates. [90,91,92,93,94]. Recently, Du et al. reported ZnO-gated hollow mesoporous silica sphere vehicles for stimuli-responsive controlled release of DOX. The ZnO quantum dot particle size is around 2–3 nm, while the average pore size of hollow mesoporous silica sphere is 2.6 nm [91]. Release profiles showed that the premature release amount of DOX is negligible, reflecting the gating effect of the ZnO quantum dots. Our research group also designed a novel supramolecular plug for controlled release of DOX (Figure 3) [94]. Ferrocene functionalized β-cyclodextrin was designed as “plugs” to achieve zero premature release. These functionalized β-cyclodextrin derivatives were anchored onto the mesoporous entrances through acid cleavable ketal linkages. When exposed to an acid milieu, this system would be activated: the acid stimulus could unplug the ferrocene functionalized β-cyclodextrins. Moreover, we also designed a sub-plug in this DDS. The ferrocene section was introduced to the C2 position of β-cyclodextrin molecules by a chemical synthesis method and it functions as a sub-plug to block the lower relative molecular weight drug gemcitabine (GEM). Without the encapsulating effect of the ferrocene section, the small cargo molecules would escape from the DDS through the β-cyclodextrin cavity. This sub-plug responds to redox potential stimulus. In this way, we realized successive release of two different drugs through two stimuli—responsive mechanisms.

The polymer type gates show advantageous satisfactory sealing effects due to the compactness of the polymer shell [86]. Besides, some biocompatible polymers such as polyethylene glycol (PEG) have the bonus effect of prolonging the circulation time and a shielding effect towards the immune system. The release function of polymer gates is usually based on a volume change, solubility change or cleavage process. Cui et al. designed a cleavable PEG gate for the controlled release in MSN-based DDSs [95]. The PEG chains were anchored on the MSNs through disulfide linkages. The amount of cargo molecules released is negligible in PBS solution (pH = 7.4). When glutathione (GSH) was introduced into this releasing system, the releasing behavior was obviously detected by UV-vis spectroscopy. With the increment of GSH concentration, the release rate was accelerated as well. Inspired by the abovementioned research, Ryu and coworkers proposed an advanced polymer gate [96]. A copolymer chain containing pyridine disulfide hydrochloride (PDS) and PEG wrapped the MSNs surface by noncovalent interaction. The self-crosslinking polymer shell could be dissembled by both dithiothreitol (DTT) and GSH, leading to DOX-releasing behavior.

The bridge type gate involves dynamic crosslinking of grafted long-chain organic molecules. The crosslinking process results in the encapsulation of inner cargo molecules. Both cleavable chemical bonds [97] and controllable supramolecular interactions [88] can be used for sealing purposes. Once the crosslinked system was interrupted, the release behavior could be initiated. Yang and co-workers [88] prepared pH and GSH dual-responsive dynamic crosslinked supramolecular networks on the outer surface of MSNs. The “bridge” in this article was constructed by grafting poly(glycidyl methacrylate) (PGMA) on MSNs and crosslinking PGMA chains through cation-dipole interactions between cucurbit[7]uril (CB[7]) and the ethanediamine (EDA) part on PGMA chains (the binding mode between CB[7] and EDA was a 2:1 host-guest complex). The pH sensitivity of this system derived from the competitive binding between the hydronium ions and EDA. When the pH ≤ 4.0, the hydronium ions disassembled the original crosslinking system by binding with CB[7]. Considering that the total bridge system was anchored onto the reservoir MSNs through disulfide linkages, this bridge gate could also be removed by the presence of glutathione. Besides, an in vitro cell-growth inhibition effect was observed by cultivating A549 cells in the presence of MSNs@DOX@CB[7]. Based on an analogous design, this research group applied a bridge type gate in layer-by-layer coated MSNs (LbL-MSNs) [98]. In this system, the macrocyclic molecule CB[7] was used to build a molecular bridge layer: the ion-dipole interaction between CB[7] and EDA realized a bridge function to connect two bisaminated poly(glycerol methacrylate) polymer (EDA-PGOHMA) layers. The self-assembly layer could be directly observed in transmission electron microscopy (TEM) images. When this dynamic supramolecular interaction was interrupted by competitive binding of hydronium ions or adamantaylamine hydrochloride (AH), the LbL structures loosened, releasing DOX molecules. The therapeutic effect of this system was confirmed by both in vitro and in vivo experiments: For dye propidium iodide (PI)-loaded LbL-MSNs, they could be quickly endocytosed in the human breast carcinoma cell line (MDA-MB-231) followed by nuclear staining, which resulted from intracellular release of PI. In vivo anticancer experiments also showed inhibited tumor growth phenomena in mice treated by DOX-loaded LbL-MSNs.

Liposome gates were reported by Brinker et al. in 2009 [99]. Noticeably, this type of gate possesses a biomimetic structure comparable to a cell membrane, providing special affinity for cellular uptake and biocompatibility. The positively charged liposome is fused onto the surface of a negatively charged mesoporous silica core to form a continuous barrier, which blocks the pre-release of inner cargos. Acidic pH values in the endosomal environment facilitate the cargo release inside a cell. Most studies concerning this type of gating strategy show a common merit: high capacity for cargo molecules [89,100]. However, there are also some drawbacks which cannot be neglected. The lipid bilayer structure is prone to disintegrate under the interruption of surface active components. So far, many efforts have been made to improve the overall function of the “protocell system”. Brinker et al. added a targeting peptide, endosomolytic peptide, fusogenic peptide and PEG to improve targeting, stability and selectivity [101,102]. Rosenholm and coworkers constructed lipid bilayer structures on hyperbranched polyethylenimine (PEI). PEI tethers the lipid bilayer close to the MSN surface and also facilitates a high loading of negatively-charged hydrophilic guest molecules [103].

### 2.2. Polymers

The research on tumor stimuli-responsive polymeric vectors has been increasingly regarded as an important area in biomedicine due to their versatile nature [24,25,26]. Usually, the polymer-based DDS have a sub-micron scale. This size promotes EPR levels. As a promising and successful platform for DDS, polymer materials exhibit major advantages such as long blood circulation, high drug loading capacity, biocompatibility and facile integration of functionalities. Up to now, many polymer materials have been proved to be biocompatible: PEG, polylactic acid (PLA), polyglycolic acid (PGA), polycaprolactone (PCL), dextrans, etc. [104]. The major loading mode is direct coverage of the drug molecules by the polymeric structure [105]. Sometimes, drug molecules are also covalently linked to a polymeric framework [106]. Inside the polymeric structure, there are a lot of repeated organic moieties, providing sufficient active sites to combine with special functional groups. With the import of these groups, polymeric materials are endowed with almost all kinds of stimuli-responsive characteristics. Apart from stimuli-responsive substances, these active sites are also designed for binding targeting agents. In this way, the pathological site recognition precision could be guaranteed. Moreover, the flexible structures of polymer materials could encapsulate solid, heterogeneous nanoparticles so that extra physical properties such as magnetism could be added [29].

Polymeric platforms are mainly built by block copolymerization. In recent years, stimuli-responsive copolymer synthesis has achieved important advances. The invention of living radical polymerization offers advantages such as mild reaction conditions, broad scope of monomers, and simple manipulation. The living radical polymerization, in particular atom transfer radical polymerization (ATRP) and reversible addition-fragmentation chain transfer free-radical polymerization (RAFT) is now widely used in the design and synthesis of polymer frameworks with different topology and stimuli-responsive functions [107,108,109]. Micelles [110], polymer conjugates [111], dendrimers [112], vesicles [113], nanogels [114], and polymer complexes [115] have all been employed as drug carriers. Upon exposure to stimuli, the polymeric framework undergoes a solubility change, collapse or swelling process. These behaviors result in controlled drug release.

Dendrimers are artificial, hyperbranched nanopolymeric architectures, with three dimensional structures. According to dendrimer-based studies, they are nanometric scale molecules, ranging from several to tens of nanometers in diameter. This well-defined size endows dendrimers with the ability to penetrate cell membranes. Dendrimers are mainly composed of three parts: inner core, branched layers (also called “generations”), and a periphery with multiple functional groups. With the accumulation of generations, the internal cavity volume increases, allowing higher drug loading capacity. Sun and coworkers built two dendrimer/lipid nanoassemblies [116]. A hydrophilic dendrimer polyamidoamine dendrimer (PAMAM) and a hydrophobic dendrimer poly(amino ester) dendrimer (PAE) were selected as drug containers. The hydrophobic anticancer drug DOX was loaded in the PAE dendrimers. Then the DOX molecules were encapsulated via lipid coating. The anticancer drug methotrexate (MTX) was loaded in PAMAM dendrimers. With the aid of the anionic surfactant sodium dodecyl sulfate (SDS), loaded PAMAM dendrimers were also covered inside a lipid layer. In the tumor extracellular environment, the DOX-PAE/lipid complex underwent cell membrane fusion and thus induced the ideal release of dendrimers. In the meantime the MTX-PAMAM/lipid was swallowed into the intracellular environment. The change of microenvironment induced disassembly of the MTX-PAMAM/lipid. In this way, two different kinds of anticancer drug were released at the tumor site, enhancing their synergistic effect.

Micelles have been developed as a novel type of drug carrier over the past few years. When the concentration of amphiphilic block copolymer molecules exceeds the critical micelle concentration (CMC), they would self-aggregate into micelles in water solution, which has emerged as a novel targeting drug delivery system with broad prospects. In aqueous medium, the micelle core is hydrophobic due to the aggregation of nonpolar groups, while the micelle shell is hydrophilic, protecting DDS from clearance by the immune system. Therefore, this system is especially suitable for stabilizing hydrophobic anticancer drugs such as camptothecin and paclitaxel. The concentration of loaded drugs is much higher than their saturation concentration in water solution. Du et al. synthesized poly(acrylamide-co-acrylonitrile)-g-PEG for building micelles [117]. The amphiphilic polymer poly(acrylamide-co-acrylonitrile)-g-PEG molecules possessed temperature sensitivity: exhibiting an upper critical solution temperature (UCST) of 43 °C. Its CMC was 30.3 mg/mL, with the concentration exceeding 30.3 mg/mL, poly(acrylamide-co-acrylonitrile)-g-PEG self-assembled into micelles. When the temperature reached 43 °C, the micelles disassembled, releasing encapsulated DOX molecules.

Vesicles are also composed of amphiphilic molecules. Unlike micelles, vesicles possess a closed bilayer structure, containing moisture inside. This enables vesicles to load both hydrophobic and hydrophilic drugs. Lee and coworkers reported an onion-like vesicle drug delivery platform with poly(ethylene oxide)-block-poly(ε-caprolactone) (PEO-b-PCL) as copolymer scaffold, differing from traditional single-walled vesicular hybrids [118]. At first, the in situ self-assembly of PEO-b-PCL resulted in nanofibrils, while the following emulsification-induced assembly generated various nanostructures, helical ribbons, tubules, and unique vesicles with high-ordered feature. Among them, the onion-like vesicles were loaded with DOX, showing high loading capacity and stepwise drug release behavior. In the intracellular microenvironment (pH 5.5), the hydrolytic cleavage of PCL resulted in the competitive dissociation of vesicles. Four stages of Dox-release were observed. This was due to a graded vesicle degradation from the outermost to the innermost layers.

**Figure 4:**
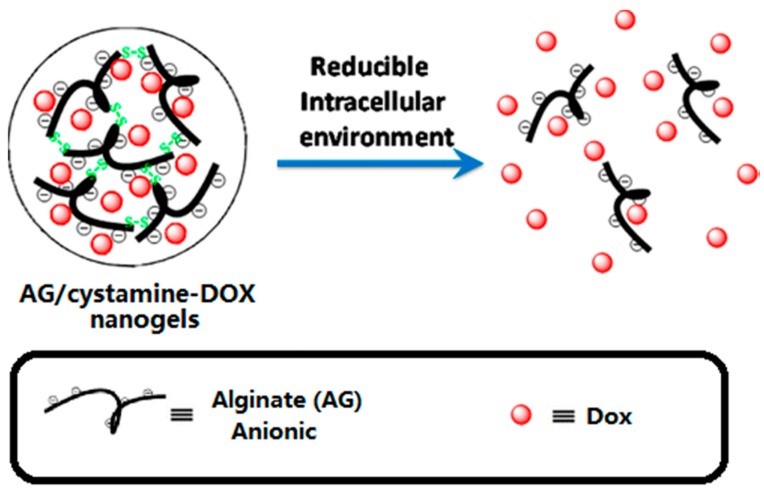
Schematic diagram of a polymer DDS. Reprinted with permission from [105]. Copyright 2013 American Chemical Society.

Hydrogels represent a class of soft materials, which possess 3D network structures containing a large fraction of water inside. At the early stage, hydrogel applications in the drug delivery field were limited because of their poor mechanical properties, and uncontrollable drug retention stability, resulting in relatively rapid release of drug molecules. Recently, researchers have introduced stimuli-responsive characteristics into hydrogel platforms, significantly increasing their potential. Tomás et al. developed a nanoscale alginate hydrogel with redox responsiveness allowing the intracellular release of DOX (Figure 4) [105]. The emulsified alginate was cross-linked with cystamine. Then DOX was loaded via electrostatic attraction. After an endocytosis process, the GSH cleaved the disulfide bonds, which disassembled the crosslinked 3D structure, releasing the DOX molecules.

### 2.3. Metal-Organic Frameworks

Metal-organic frameworks (MOFs) are built by coordination interactions between metal cations and organic ligands, presenting a coordination network structure in one-, two-, or three-dimensions [22]. Since the discovery of MOFs, this new class of hybrid organic-inorganic materials has grown rapidly, making great process in the fields of gas storage [119], catalysis [120], separation [121], electronics and optical applications [122]. Early studies of MOFs in drug delivery applications focused on the storage and transmission of gaseous drug molecules such as nitric oxide [123]. As research continued, researchers have revealed that the pore size could be tuned and their chemical modification process is facile. These two characters make MOFs a versatile platform for theragnostic devices.

In 2009, Boyes et al. developed a multifunctional nanoscale theragnostic device with a MOF as scaffold [19]. In this research, vacant orbitals on gadolinium ions (Gd^3+^) on the outside surface of a Gd MOFs’ surface were utilized to link end the group thiolates of biocompatible polymer chains of poly(*N*-isopropylacrylamide)-co-poly(*N*-acryloxysuccinimide)-co-poly(fluorescein *O*-methacrylate). The copolymer chains contained a fluorescent *O*-methacrylate (FMA) tagging section. Also, the *N*-acryloxysuccinimide (NAOS) section works as active site to tailor targeting ligands and antineoplastic drugs like MTX. When MTX-containing polymer-modified Gd MOFs were incubated with FITZ-HSA tumor cells, the results showed decreased cell viability which was in accord with the cell viability results obtained using free MTX. The authors postulated that the attached MTX on the copolymer chains have same overall function compared to free MTX.

Recently, Yang and co-workers constructed supramolecular switches on MOF surfaces, forming targeted drug release systems [124]. In this research, the [2]pseudorotaxane gatekeepers were constructed with positively charged pyridinium (Py) stalks and negatively charged carboxylatopillar[5]arene (CP5) via host-guest complexation. This design successfully introduced a pseudorotaxane type gate into the MOF nano-platform. Release behavior was triggered by acid activation and competitive binding: upon lowering the pH, CP5 sodium salts gradually protonated and precipitated out, which caused the disassembly of the original gatekeeper. When introducing viologen molecules into the DDS environment, CP5 dethreaded from the Py stalks and bound with viologen whose binding affinity is higher than that of Py. By using a similar protocol, Yang et al. developed stimuli-responsive supramolecular gates on the zirconium (Zr)-based MOF UiO-66-NH_2_ for central nervous system disease therapy [20,21].

### 2.4. Quantum Dots

Quantum dots (QDs) are composed of several hundred or thousand atoms; therefore they are extremely small nanoparticles, with an average radius between 1 to 10 nm [125]. This compact size results in quantum confinement effects: the continuous energy-band structure is restricted into split energy-level structures [126]. Based on this character, QD materials could be excited to emit fluorescent light, therefore, this newly-emerged material shows major advantages in fluorescent imaging for medicine diagnostic purposes [127,128]. Besides, the multiple surface modification strategies further expand their application range. However, traditional QDs materials (such as CdSe QDs, CdS QDs) have an underlying liability that limits their medical application: their long term potential toxicity. Although passivation methods such as appropriate surface coatings could limit the toxicological and pharmacological effects of QD materials, researchers have recently turned attention to new QD materials.

One way of introducing QDs into a DDS is to incorporate them into larger drug delivery vehicles; therefore, the tracing function brought by QDs enables the study of biodistribution, compatibility, pharmacokinetics and pharmacodynamics, etc. As mentioned before, QDs materials could be used as “plug” gates in MSN-based DDS [90]. Recently, Kong et al. developed ZnO@polymer complex DDS, presenting a core–shell structure [129]. The ZnO QDs synthesized by a two-step copolymerization method could be stably dissolved in water, while also exhibiting photoluminescence stability. Moreover, the photoluminescence emission band of DOX was also strengthened due to the fluorescence resonance energy transfer effect from the ZnO QDs to DOX molecules. The release process was turned on by acid stimulus: the ZnO QDs would decompose in an acidic environment (pH ≤ 5). Correspondingly in confocal laser scanning microscopy images, a DOX red fluorescence signal was detected in lysosome positions. Free zinc ions were also found in the intracellular environment using a zinc-specific fluorescent dye as probe. With the accumulation of detection time, the zinc ion signal was enhanced. These results revealed the DOX loaded ZnO@polymer QDs were firstly engulfed, then the QD scaffolds were decomposed at lysosome positions to release DOX molecules.

Since their discovery in 2004, carbon quantum dots (CQDs) have exhibited excellent biocompatibility and are generally recognized as powerful platforms to build safe, multifunctional DDSs. In 2013, quinoline-chlorambucil units (Qucbl) were covalently linked with CQDs by Singh and coworkers [68]. Inside the Qucbl unit, the chlorambucil section works as active anti-tumor component and the quinoline section can be photo-cleaved by both one-photon excitation and two-photon excitation mechanism under UV irradiation to release chlorambucil molecules. The Qucbl modified CQDs exhibited a tunable emission spectrum (350–450 nm) and relatively high fluorescent quantum yields. The photorelease behavior was detected under UV light irradiation, while very low drug release was observed under a He–Ne laser light source. Versatile surface modification methods enrich the on-demand drug release types of QD-based DDSs: in addition of light irradiation, redox [130] and acid [131] stimuli-responsive mechanisms were also developed.

### 2.5. Carbon Nanotubes

Carbon nanotubes (CNTs) have been identified as an important member of the carbon-based nanomaterial family, since their first discovery in arc-burned graphite rods in 1991. The past two decades have witnessed the evolution of CNTs into versatile platforms for sensors, optics, electronics and gas storage and biomedical applications [132,133,134,135,136]. This is because CNTs possess marked benefits such as strong and stiff mechanical character, large surface area, and photoluminescence. These highly attractive features make CNTs a promising drug delivery vehicle. For example, owing to the high surface area to volume ratio (theoretically 1300 m^2^/g), CNTs demonstrate immense potential for extensive loading of anti-cancer drugs. On the sidewalls of CNTs, vast amounts of drug molecules are bound either covalently or non-covalently. The intrinsic optical properties of CNTs could also be used for biomedical imaging, which reflects the bio-distribution of CNT-based DDS.

CNT materials exist in two dominant forms: single-walled CNTs (SWCNTs) and multi-walled CNTs (MWCNTs). The categorization is based on the number of graphene layers. The structure of SWCNTs can be conceptualized as a one layer cylinder of carbon atoms. This unique structure of SWCNTs facilitates transmembrane penetration, realizing direct translocation through cellular membranes. A simple SWCNT-based stimuli-responsive DDS was recently developed by Kang and coworkers [137]. In this system, chitosan coated SWCNTs were first covalently conjugated with fluorescein isothiocyanate (FITC). The FITC served as indicators for tracking the location of the nanotube carriers inside cells. Then the anti-cancer drug DOX was attached onto the sidewall of the SWCNTs by means of π–π stacking functions. Confocal image results demonstrated that these SWCNT-based DDS were able to undergo fast cell internalization. After the endocytosis process, the acidic environment in the lysosomes ionized DOX, which changed the interaction between DOX and SWCNTs, inducing DOX release inside cells. Despite the abovementioned benefits, the extension and application of SWCNT-based DDSs is still limited by their inherent drawback: poor dispersion in aqueous solution. To overcome this drawback, a PEGylation strategy is often used to improve the affinity of SWCNTs for water solutions. Apart from this method, recently, a novel mesoporous silica coated SWCNT (SWCNTs@mSiO_2_) structure was developed in collaboration with Liu and coworkers [138]. This composite structure was also decorated by PEG chains to enhance the solubility and stability under physiological conditions. Unlike other SWCNT-based DDSs, the anti-cancer drug was stored in the mesoporous silica layer, while SWCNTs played the core role. This system converted near infrared (NIR) energy to heat, promoting photothermally accelerated cellular uptake of nanocarriers and light-triggered drug release inside cancer cells. Release of DOX from SWCNTs@mSiO_2_ was observed when irradiated with NIR irradiation. In addition to its drug delivery function, due to the strong NIR absorbance ability of these SWCNTs, this system also worked as a multimodal imaging probe, revealing the accumulation efficiency of SWCNTs@mSiO_2_ in the bodies of mice after intravenous injection.

MWCNTs they consist of multiple rolled graphene layers, offering ultrahigh surface areas. In recent years, research concerning MWCNT-based DDSs has focused on the functionalization of MWCNTs to solve their problems such as poor specificity, non-biocompatibility and highly toxicity. In a study by Li et al., MWCNTs were dual-functionalized using folic acid (FA) and iron nanoparticles [139]. This nanodevice for DOX delivery is capable of magnetic localization (passive targeting) and FA targeting (active targeting). As a result, the nanocarriers would be firstly enriched around tumor sites, and then through biological affinity interaction between the FA and the over-expressed FA receptor, cancer cells would be recognized by the nanocarriers. The DOX release process was assisted by near infrared radiation (NIR). Because of the incorporation of these two targeting agents, the drug delivery efficiency was greatly enhanced. In another study, Datir et al. conjugated hyaluronic acid with MWCNTs, which facilitated their internalization into lung cancer cells via hyaluronic acid receptor-mediated endocytosis [140]. After being endocytosed, this platform exhibited a pH-responsive drug release behavior: the low pH environment inside cancer cells destroyed the π–π stacking function between DOX and MWCNTs. With this high specificity advantage, the nanocarriers displayed higher apoptotic activity than free DOX under equivalent concentrations.

## 3. Stimuli-Responsive Mechanisms

### 3.1. Intrinsic Stimuli

In this section, we will examine recent progress in pH, redox, biomolecules related DDSs. These three categories are the most frequently studied as the major intrinsic stimuli.

#### 3.1.1. pH-Responsiveness

pH responsiveness used for drug delivery field generally refers to a low pH stimulus. There exists a natural pH gradient in different parts of the human body, for example, the pH environment in the digestive system is much more acidic than in other organs. Among these pH gradients, the difference between tumors and normal tissues offers a great platform to design stimuli-responsive drug release mechanisms [141]. Warburg found that the rapid proliferation effect in tumor tissues results in enhanced glycolysis and excessive accumulation of lactic acid. The Warburg effect causes a pH decrease in the cancerous environment, deviating from the normal physiological environment pH value (about 7.4).

The transition from a neutral milieu to an acidic milieu could lead to significant changes in materials’ structure and performance. Chitosan-based drug delivery materials can undergo swelling processes and release drug molecules. Metal oxide nanoparticles would dissolve in an acidic milieu. Some specific materials such as carboxylatopillar[5]arene would precipitate out in acidic solution. These transformations can be summed up in three major types:

1. Acid-caused charge reversal;
2. Acid-caused dissolution behavior change;
3. Acid-caused hydrolysis reactions and bond breaking.

In 2016, Zhao et al. studied the charge conversion character of PEG-functionalized poly-allyamine (PEG-(PAH/DMMA)), finding that when going from normal physiological conditions to the mildly acidic tumor extracellular microenvironment, the charges of PEG-(PAH/DMMA) changed from negative to positive [142]. This is caused by exposure and protonation of the amino groups within the polymer molecules. The release mechanism is based on the acid-caused charge conversion: anti-tumor drugs, like carbon dots-based cisplatin (IV) were originally loaded into PEG-(PAH/DMMA) via electrostatic forces. Once the charge of the carrier material turned positive, the formal attractive interaction changed to a repulsive interaction which facilitates the release process. The extra beneficial effect of this charge conversion is that the original negative state helps to prolong the DDS circulation time within the human body, avoiding rapid clearance by the immune system. Also, after the charge conversion process, positively charged carbon dots-based cisplatin (IV)@PEG-(PAH/DMMA) absorbed onto the negatively charged cancer cell membranes more easily, which further promotes cellular uptake. Based on the same mechanism, Zhang et al. developed pH sensitive micelles for delivering DOX [143]. In the first step, the polypeptide formed positively charged micelles. Then the micelle scaffold was modified with TAT peptide and the surface charge was turned to negative. After loading DOX, when exposed to the mildly acidic tumor extracellular microenvironment, amide hydrolysis made the integral DDS switch to positive charged again. The charge reversal enabled the DDS to be quickly internalized by tumor cells. Inside the acidic endo/lysosomes (pH ≈ 5.0) of tumor cells, the nuclei targeting agent TAT peptide was activated. Eventually the nuclear drug delivery was achieved.

Pillararene belongs to a novel type of macrocyclic molecules. The symmetrical and rigid structure endows it with special host-guest properties in supramolecular chemistry. After carboxyl modification, the pillararene derivative, carboxylic water soluble pillararene (WP) exhibits great potential in medical applications. In acid solution, the protonation process of carboxyl groups makes WP molecules precipitate out. Du et al. designed a WP-based supramolecular valve was applied onto the orifices of MSNs [144]. The carboxylic water soluble pillar[6]arene (CPA[6]) stayed on the guest stalk, playing a blocking role. In acidic buffer solution that simulates the cancer intracellular microenvironment, the encapsulated cargo molecules would be released because the precipitated CPA[6] molecules lost their blocking function. Following the same mechanism, Wang et al. developed WP-based supramolecular vesicles for controllable drug release [145]. In aqueous solution, a water-soluble pillar[5]arenes (WP5) firstly combines with a long chain lysine derivative, resulting in a 1:1 complex. Then the host-guest complex would self-assemble into vesicles. The drug-containing vesicles are disassembled in acidic solution, accompanied by the disappearance of the Tyndall effect. The acid-caused precipitation of WP5 molecules is the reason behind the acid stimulated drug release.

pH-Cleavable bonds also serve as an important pH-responsive mechanism. Anti-tumor drugs could be linked onto carrier materials via these pH-cleavable bonds, or the blocking entity could be removed through hydrolysis of pH-cleavable bonds. So far, hydrazone, ketal and amido bonds have been used in designing pH-responsive DDSs. Our research group recently fabricated a dual-pH responsive supramolecular valve on hollow zirconia nanospheres. This valve is composed of the macrocyclic molecule cucurbituril[7] and the stalk molecule propanone bis(2-aminoethyl) ketal (PBAEK) [34]. The PBAEK units contain ketal bonds, which are broken due to the acid hydrolysis reaction. With the breakage of PBAEK units, the supramolecular valve disintegrated, resulting in release of DOX molecules in acidic aqueous solution. Zhang et al. prepared DOX- containing supramolecular microcapsules for tumor chemotherapy [32]. The DOX molecules were covalently linked with adamantane (Ad) via acid-cleavable hydrazone bonds. Then the DOX-Ad complex was loaded onto the walls of microcapsules by host-guest interaction. The release behavior was triggered by acid stimulus: at pH 7.4, a negligible amount of DOX was detected, while at pH 5.5, the hydrolysis of the hydrazone bond between the AD moiety and DOX freed the DOX molecules. In vitro cell experiment results showed a pH-dependent cancer cell growth inhibition effect.

#### 3.1.2. Redox-Responsiveness

Apart from the pH-responsiveness mechanism, the redox-responsiveness mechanism is another frequently adopted strategy for fabricating stimuli-responsive drug delivery vehicles. Reducing substances such as vitamin C (ascorbic acid), vitamin E and glutathione (GSH) are widely distributed in the human body. Among them, reduced state GSH plays a key role in the human metabolism process. It is reported that the tumor intracellular concentration of GSH is about 2–10 mM, far exceeding that in extracellular environment (by almost a thousand-fold) [146]. Due to its reducibility, GSH mediates disulfide bond cleavage reactions, through a dithiol-disulfide exchange process. Upon cleavage, it may lead to the disassembly of polymeric backbone materials or gatekeeper removal on mesoporous materials.

In regard of polymer disassembly, Farokhzad et al. designed polymeric nanoparticles of l-cysteine-based poly(disulfide amide) (Cys-PDSA) for delivering docetaxol [147]. With the introduction of disulfide bonds, this DDS was endowed with tunable biodegradability properties. After GSH treatment, a cargo molecule release profile was evident. In the meantime, reduction of the polymer molecular weight also proved the disassembly of carrier materials. Besides, through tailoring the carbon chain length of fatty diacid units, the degradation rate could be regulated, making spatiotemporal drug delivery possible. As confirmed by cell experiments, this is an auspicious candidate for combination therapy. According to Balendiran et al., the GSH level inside normal cells is also higher than in the extracellular microenvironment [144]. To avoid unwanted uptake by normal cells, a targeting function is needed for improving the overall therapeutic efficiency. Zhu and coworkers prepared redox-responsive hyperbranched polymers with the DNA aptamer AS1411 as targeting agent to mediate the endocytosis process [148]. The polymer backbone was synthesized via RAFT polymerization and self-condensing vinyl polymerization (SCVP), leaving vinyl moieties as terminal groups to be functionalized with AS1411. This amphiphilic polymer assembled into nanoparticles, generating a hydrophobic inner core to accommodate DOX molecules. After drug loading, this system was treated with 10 mM GSH, which simulated the cytoplasmic environment. DLS results showed decreased nanoparticle diameter, while for the sample which was not GSH-treated, the DLS results did not changed remarkably. This indicates that GSH cleaved the disulfide bonds in polymer chains, contributing to the breakage of the polymeric nanoparticle carriers. This was accompanied by the disentanglement of the hyperbranched structure, and released DOX molecules were detected in the cytoplasm of MCF-7 cells under confocal laser scanning microscopy, resulting in apoptosis.

In addition to the polymeric materials, mesoporous structured materials could also be modified for redox-responsive drug release by introducing S–S bonds into linked gate keepers. Zhao et al. designed multifunctional MSNPs: the disulfide bonds linked β-cyclodextrin with the MSNP surface. The β-cyclodextrin rings function as gatekeeper to block DOX molecules. The PEG derivatives mPEG-Ad and FA-PEG-Ad were further anchored on MSNPs via host-guest interaction between β-cyclodextrin and adamantane units [149]. The PEG chain could prolong the blood circulation period of MSNPs, avoiding premature clearance. The folic acid unit on PEG chains provided a targeting function, leading the DDS to the peritumoral environment by recognition of the folate receptor which is overexpressed on the surface of tumor cells. Based on an analogous design, this research group fabricated another hollow mesoporous silica nanoparticle-based drug delivery vehicle with redox-responsive character [64]. In this case, adamantane molecules were linked onto the surface of hollow mesoporous silica nanoparticles via disulfide bonds. Then a macrocyclic molecule, lactobionic acid-grafted-β-cyclodextrin, was capped on the adamantane units through a strong supramolecular complexation force, entrapping DOX molecules. The lactobionic acid modified on β-cyclodextrin also works as targeting ligand. The drug release behavior in neutral Tris buffers was very limited, and only negligible amounts of DOX were detected. After treatment with 10 mM GSH, the DDS was able to discharge DOX, showing an accelerated release rate and long-term release behavior. This was due to the cleavage of S–S bonds, removing the capping supramolecular complex.

Recently, Crespy and coworkers designed a novel type of redox-responsive release system, by encoding molecules in the shell of polymeric nanoparticles through disulfide bonds [150]. The thiol group in mercaptobenzothiazole (MBT) molecules could be transformed to disulfide bonds through dithiol–disulfide exchange. The researchers encoded MBT molecules as side chains of the copolymers. On exposure to the reducing agent tributylphosphine (TBP), which plays a similar role as GSH, the disulfide bonds between the polymeric backbone and the MBT section were cleaved. Furthermore, with the removal of MBT, the backbone of the hollow structured polymeric nanoparticles was also damaged. This hollow structure was originally loaded with hydrophobic cargo molecules. Therefore, two kinds of payload could be released by a single redox stimulus. Although this release system is not currently used for medical purposes, this idea inspired us to think that thiol-containing drug molecules could be designed for direct linking via disulfide bonds.

Recently, oxidizing substances were also studied as triggers, developing into a new research focus. According to up-to-date study results concerning the biological features of cancer cells, the reactive oxygen species level is very high in cancer cells compared to normal cells. Therefore, it is possible to build a stimuli-responsive mechanism based on oxidizing substance triggers. As the main component of the intracellular oxidate, hydrogen peroxide is involved in many tumor metabolic processes: the variability of gene, proliferation and apoptosis. In tumor tissues and inflamed tissues, there are accumulations of H_2_O_2_.

Almutairi and co-workers reported a H_2_O_2_-degradable polymer which could be used as scaffold of oxidate-responsive DDS [151]. Boronic ester derivatives were copolymerized with adipic acid to synthesize a H_2_O_2_ reactive polymer. This polymer material exhibited a nanoparticle structure. The interior core could accommodate hydrophobic cargo molecules. Upon exposure of H_2_O_2_, quinone methide rearrangement was initiated, resulting in the hydrolysis of boronic ester units and degradation of the nanoparticle carrier. The degradation rate could be further mediated by changing the linkage between the polymeric scaffold and boronic ester units. Cargo release behavior was detected by fluorescence intensity monitoring: the wavelength migration and fluorescence quenching of Nile Red confirmed the movement of cargos from the hydrophobic inner core to a more polar medium. Besides, the degraded polymer structure could also be precisely observed in TEM photos. Xia et al. also prepared a reactive oxygen species (ROS)-responsive delivery system for gene transmission to cancer cells [49]. Based on the same design concept, thioketal linkages were adopted as ROS-cleavable units in the polymeric structure. The H_2_O_2_ cleavage process broke the thioketal units, generating acetone. GRP78-binding peptide was further conjugated with polyplexes, functioning as targeting agent.

#### 3.1.3. Biomolecule-Responsiveness

The development of biomolecular chemistry has revealed their biodistribution within the body, and their role in biochemical reactions. Intensive understanding of biomolecules fills the void in biomolecule-related drug delivery. In this field, enzyme-sensitive DDSs are the most developed system. As major biocatalytic substances, enzymes are involved in many biochemical reactions within the body. For example, esterases catalyze ester hydrolytic reactions; glycosidases catalyze the hydrolysis of glycosidic bonds; peptidases catalyze the hydrolytic breakdown of proteins into amino acids; and reductases catalyze hydrogenation reactions. Noticeably, enzymes contribute to the biodegradation of biomacromolecules and some specific enzymes are over-expressed in cancer cells, indicating great potential for developing enzyme-sensitive DDSs.

Máñez et al. prepared poly-l-glutamic acid (PGA)-gated MSNs for pronase-responsive drug delivery [54]. Upon exposure to pronase, PGA gates were removed, releasing DOX molecules. The performance of this DDS in a cellular context also showed valid cellular uptake and enzyme-caused SK-BR-3 cell apoptosis. Huang et al. also introduced an enzyme involved release mechanism into DDSs. The biocompatible copolymer backbone *N*-(2-hydroxypropyl) methacrylamide (HPMA) was adopted as drug carrier [52]. Model drug F8A (H1) peptide and targeting peptide were conjugated to the polymer scaffold via lysosome enzyme degradable glycylphenylalanylleucylglycine (GFLG) linkages. After specific tumor cell uptake, the GFLG linkages were cut off with lysosome enzyme, unleashing subunits composed of drug peptide and targeting peptide. Drug accumulation at the nucleus could be visualized in confocal laser scanning microscopy photos.

Recently, ribonucleic acid-related compounds were also confirmed as effective internal triggers for on-demand drug release. Xu and co-workers constructed oligonucleotide pair gates onto the orifices of MSNs [53]. The oligonucleotide pair was made up of 7-amino-4-methylcoumarin (AMCA) dye terminated short oligonucleotide (Flare_A_) and FITC labeled long oligonucleotide (S1_F_). The fluorescence resonance energy transfer (FRET) pair AMCA-FITC constituted a real time monitoring mechanism. Exposure to survivin mRNA induced the dehybridization between S1_F_ and Flare_A_. The FITC emission band (520 nm) showed an obvious decrease because the FRET pair also disassembled. In the meantime, an enhanced DOX emission band (450 nm) was detected. In vitro experiment results also confirmed the efficiency of the survivin mRNA responsive mechanism.

### 3.2. Extrinsic Stimuli

In this section, we will discuss man-made stimuli-responsive mechanisms. These stimulus signals did not originally exist inside the human body. Researchers have developed these new methods to achieve more accurate control over drug release behaviors.

#### 3.2.1. Thermo-Responsiveness

Recently the combination of hyperthermia treatment and chemotherapy showed significant curative effect improvements. On the one hand, localized hyperthermia helps kill cancer cells by putting the tumor tissues under a high temperature environment (42.5–43.5 °C). On the other hand, the thermal stimulus could dilate vessels and change the penetrability of tumor cell membranes, promoting delivery of anti-tumor drugs. In the field of stimuli-responsive DDS, thermo-responsiveness was also developed as an effective method, optimizing the overall efficiency of thermotherapy and chemotherapy. Photothermal transformation and magneto-calorification effects could be used as thermal sources. Temperature-sensitive properties were found in some specific polymer materials such as poly(*N*-isopropylacrylamide) (PNIPAM). These temperature-sensitive polymers have a lower critical solution temperature (LCST) parameter: when the ambient temperature is lower than the LCST, the polymer material manifests a water soluble property swelling state due to the hydrogen bonds between polymer chains and water molecules. The disruption of hydrogen bonds happens as the temperature increases, resulting in insolubility and collapse of the temperature-sensitive polymer. This phase transition behavior could be developed into an block-open mechanism in DDS.

Recently, Yu et al. prepared a thermo-responsive DDS with PNIPAM. Together with Fe_3_O_4_ nanoparticles, the anticancer drug 5-fluorouracil was encapsulated in PNIPAM [36]. This complex was further covered by a mesoporous silica shell with a chitosan-rhodamine 6G layer. At 25 °C, the release of 5-fluorouracil was much slower than that at 45 °C. This was due to the shrinkage of PNIPAM that squeezed out the loaded 5-fluorouracil. However, the LCST of unmodified PNIPAM is merely 32 °C in water, which greatly limits its in-vivo applications because the normal temperature of the human body is about 37 °C. A thermo-responsive DDS with a trigger temperature lower than 37 °C could suffer pre-leakage of drug molecules. Researchers found that copolymerization with a hydrophilic monomer increases the LCST, while copolymerization with a hydrophobic monomer decreases the LCST. To solve this problem, Ren and coworkers prepared the thermo-responsive star-block copolymer 6sPCL-bP(MEO_2_MA-co-OEGMA), which was prepared by ATRP with bromo-poly(ε-caprolactone) (6sPCL-Br) as macroinitiator, 2-(2-methoxyethoxy)ethyl methacrylate (MEO_2_MA) and (ethylene glycol)methacrylate as monomer. The LCST could be precisely controlled by changing the ratio of MEO_2_MA/OEGMA [37]. They found that when the molar ratio of MEO_2_MA/OEGMA was 92:8, the trigger temperature of this system was optimized to 43 °C, meeting the requirement for in vivo applications. Mn, Zn-doped ferrite magnetic nanoparticles (MZF-MNPs) were also introduced into the polymeric micelles to function as heaters under an alternating magnetic field (AMF).

Temperature gradients can also induce other physical and chemical properties. Yang et al. developed a Ca^2+^, pH and thermo triple-responsive DDS [20]. A CP5-quaternary ammonium salt supramolecular valve was constructed to encapsulate 5-fluorouracil into the pores of Zr-MOFs. For thermo-responsive release, hyperthermia (60 °C) weakened the supramolecular interaction between CP5 and the quaternary ammonium salt stalk. Sung et al. constructed a thermo-responsive bubble-generating liposomal system for delivery of the drug DOX [35]. Ammonium bicarbonate was encapsulated as a thermo active site into liposomes. When the ambient temperature exceeded 42 °C, it underwent a decomposition reaction (NH_4_HCO_3_ → NH_3_(aq) + H_2_O(l) + CO_2_(g)). CO_2_ gas overflowed from inside the liposomes in the form of bubbles, destroying the integrity of the liposomal carrier, thereby releasing the loaded DOX molecules under thermal stimulus.

#### 3.2.2. Light-Responsiveness

Light triggers remain among the most popular stimuli-responsive mechanisms, due to their non-invasive nature [152]. The major advantage of light-responsive DDSs is their temporal and spatial controllability: the drug releasing behavior can be precisely controlled by applying specific light irradiation at a specific position. Lights with different wavelengths possess different properties. So far, DDSs that respond to ultraviolet (UV) light, visible light, and near-infrared (NIR) light all have been developed and applied. The light-actuated DDS usually work based on three main mechanisms: light-induced isomerization, bond cleavage and disaggregation of carrier materials.

The wavelength of ultraviolet (UV) light is in the range of 10–400 nm, shorter than that of visible light but longer than X-rays. It is well known that azobenzene, coumarin, spiropyran, pyrenylmethyl, *o*-nitrobenzyl and coumarin groups are UV light-responsive chromophores. UV light possesses high energy to trigger photochemical reactions. For azobenzene and its derivatives, the azobenzene unit mainly exists in the *cis* form under UV light and presents the *trans* form under visible light. This UV-induced isomerization enables azobenzene to work as a “stirrer” and blocking agent. Coumarin and its derivatives undergo reversible [2+2] photodimerization with the aid of UV light. The photodimerization results in the combination of two coumarin units, which is usually designed as a photo-sensitive bridge gate on MSN scaffolds. Spiropyran and its derivatives undergo a reversible transition from the merocyanine form (under UV light) to the spirocyclic form (under visible light). The charge distribution and hydrophilicity properties of these two forms are quite different, providing possibilities to construct stimuli-responsive DDSs. Coumarin-, *o*-nitrobenzyl- and pyrenylmethyl-based molecules are UV-cleavable. By introducing these units into a light-responsive DDS, we can control the integrity of the carrier scaffold and their drug molecule releasing behavior.

Lu and coworkers constructed an azobenzene-containing “gate keeper” onto β-cyclodextrin modified hollow mesoporous silica nanoparticle surfaces [153]. Azobenzene units were grafted into the amphiphilic polymer poly(PPHM-co-PEGMEM) via copolymerization. The on/off switch function was realized by UV irradiation. The *trans* form of azobenzene could be recognized by β-cyclodextrin through host-guest interaction while the *cis* form could not. Therefore, when exposed to a UV stimulus, the azobenzene units detached from the β-cyclodextrin cavities, removing the protective polymeric gate keepers. Ibuprofen (IBU) molecules then escaped from the mesoporous channels. Under conversion with UV light and visible light, the cargo molecule release profile presented an on/off mode. Based on a similar design concept, Wang et al. designed a UV-sensitive complex between water-soluble pillar[6]arene (WP6) and azobenzene derivatives. The amphiphilic supramolecular complexes self-assembled into vesicles in water solution [154]. Upon applying UV irradiation, photoinduced *E*/*Z* isomerization attenuated the binding affinity between the azobenzene guest and WP6 host, leading to disassembly of the supramolecular complex and eventually the disintegration of the vesicle structure. Therefore, the hydrophobic anticancer drug MTX was released from the cavity of vesicles. Besides, due to the acid-responsiveness of WP6, MTX would also be released when the DDS was exposed to an acidic environment.

However, due to that the fact human tissues strongly absorb UV light, the penetration ability of UV light is relatively low and usually, the penetration depth is no larger than 10 mm. Besides, UV light possesses relatively high energy, which has been proven phototoxicity by destabilizing cells. Recently, researchers have turned their attention to a safer light source. Near-infrared (NIR) light refers to the light with wavelength between 780–2526 nm. The development of two photon excitation shed new light on NIR light irradiation applications in controlled delivery processes. Compared to UV light, NIR light possesses advantages such as an absence of side effects and high penetration ability (on the centimeter scale). Besides, NIR light could be converted into thermal energy by noble metal nanoparticles, further enlarging its clinical application field. Recently, with the development of up-conversion nanomaterials (usually lanthanide-based substances), the photon energy could be elevated from NIR region to UV-vis wavelengths. This enables NIR to participate in photoresponsive chemical reactions [155].

Zhang and coworkers prepared a NIR triggered liposome DDS [156]. Inside this NIR responsive DDS, the upconversion nanoparticles (UCNPs) Tm,Yb@NaGdF4 were encapsulated by covering UCNPs with amphiphilic 1,2-distearoyl-sn-glycero-3-phosphocholine (DSPC) phospholipids. Under excitation with 980 nm NIR irradiation, UV-vis light is emitted. The upconverted UV-vis light promoted the continuous rotation-inversion movement of azobenzene units, destabilizing the lipid bilayer structure. Upon applying pulsed NIR irradiation, a stepwise release profile emerged, proving the efficiency of NIR responsiveness.

#### 3.2.3. Magnetic Field Responsiveness

As a non-invasive signal source, magnetism has attracted great interest in developing advanced DDSs. At the early stage, magnetism was introduced into DDSs to provide control over DDS movement by applying an external magnetic field. Magnetic nanoparticles such as Fe_3_O_4_ nanoparticles also work as contrast agents, playing an important role in magnetic resonance imaging diagnosis. Lately, DDSs that respond to magnetic fields have been successfully developed. Magnetism features are no longer confined to auxiliary functionalities in cancer treatment. Tseng et al. embedded magnetic nanoparticles (adamantane-grafted Zn_0.4_Fe_2.6_O_4_) into DOX-encapsulated supramolecular nanoparticles which were composed of polymeric blocks (β-cyclodextrin modified PEI, adamantane modified PEG, adamantane modified polyamidoamine) [157]. The magnetic nanoparticles converted AMF into heat. Because the self-assembly of supramolecular hydrogel network is driven by noncovalent π–π stacking interactions, this dynamic crosslinking system could be disturbed under AMF-induced heat, releasing encapsulated DOX molecules. As the result of AMF pulsing, the drug release profile exhibited a stepped feature. After the optimization of the control parameters (DDS size and AMF intensity), effective tumor suppression was realized for in vivo experiments.

#### 3.2.4. Ultrasound Responsiveness

Ultrasound also belongs to the non-invasive stimulus category. Like magnetism, ultrasound was originally developed as a clinical diagnostic imaging method. Recent evidences have proved that high-frequency sound waves can damage some specific kinds of DDS scaffolds (such as liposomes and micelles). Besides, along with the improvement of sonochemistry, researchers also found that ultrasound could induce chemical reactions, being even able to cleave some chemical bonds. Vallet-Regí and coworkers prepared an ultrasound responsive gatekeeper on a MSN surface [158]. The polymeric gate contained an ultrasound-responsive monomer, 2-tetrahydropyranyl methacrylate (THPMA). This unit was originally hydrophobic; after ultrasound exposure, the acetal group was cleaved, generating a hydrophilic product, methacrylic acid (MAA). Through hydrophobic-hydrophilic transformation, opening and closing of the gates could be achieved: the main framework of the polymeric nanogate was thermoresponsive poly(2-(2-methoxyethoxy)ethyl methacrylate). When the outer temperature was lower than the LCST, the polymer chains presented a coil-like conformation, allowing the cargo molecules to be loaded in the mesopores. While when the outer environment temperature was higher than the LCST, the collapsed polymer chains played a blocking role to prevent cargo release. With the application of ultrasound, the hydrophobic THPMA units turned into a hydrophilic MAA, enhancing the overall solubility of the polymeric nanogate. The dissolved polymer gate lost its blocking function, causing ultrasound-induced drug release behavior.

## 4. Clinical Development

The marriage of pharmacy and nanotechnology has fueled the development of nanomedicine, revolutionizing chemotherapy at both the research and clinical levels. Since Bangham proposed his theory of nanoparticle-based drug delivery systems in the 1960s, a multitude of nanoscale drug carrier platforms have been reported, leading to an enhanced performance in both in vitro and in vivo experiments. In the clinical practice aspect, the 1990s witnessed the appearance of the first generation of clinically approved nanoparticle-based drug delivery systems for cancer therapy. As an important representative of them, Doxil^®^ was applied to curing human immunodeficiency virus (HIV)-related Kaposi’s sarcoma, ovarian cancer and breast cancer. This nanomedicine encapsulated DOX inside a liposome scaffold, and had lower dose-limiting cardiotoxicity compared to the small molecule counterpart. Theoretically speaking, when formulated correctly DDS shows advantages include: (1) fine-tuning and optimization of drug concentrations within therapeutically appropriate ranges at tumor locations; (2) controllable release site and drug release rates over a prolonged period of time; (3) improved therapeutic index of hydrophobic drugs through encapsulation; (4) protection of anti-tumor drugs from rapid clearance by the mononuclear phagocytic system; (5) mitigation or elimination of side effects and toxicity of the delivered drugs.

However, up to now, only a handful of anti-cancer nanocarrier systems have passed FDA clinical trials for commercial application and their major achievement has been confined to alleviation of systematic toxicity; in other words, their huge potential in improving overall therapeutic effectiveness is still far from being a reality. Most DDS-based studies continue to be in a conceptual phase and the information about in vivo nanocarrier behavior is mainly collected from animal experiments. The reasons why these advanced DDS have yet to be full applied were not only because of the expensive, time-consuming review process. They also include: (1) insufficient biological understanding of DDS behavior at the cellular, organ and body levels; (2) difficulty in scalable manufacturing; (3) inadequate effective screening technology; (4) economic and financial barriers.

The current mainstream of applied DDSs are mainly divided into three categories: liposomes, protein nanoparticles and polymeric nanoparticles. Table 2 displays some representative examples of them. Among them, the most fully-developed platform is liposomes. As the first generation DDS platform, liposomes were considered excellent therapeutic carriers. The unique structure of a hydrophobic core and hydrophilic shell improves the biocompatibility, pharmacokinetics and biodistribution of hydrophobic anti-cancer drugs. With a further pegylation process, the blood circulation time of liposome-based DDSs could be significantly enhanced. In the protein nanoparticle platform, the first commercial product Abraxane^®^ is composed of albumin and paclitaxel, a hydrophobic taxane drug. Through reversible noncovalent binding, paclitaxel was loaded into the albumin carrier, which is approximately 130 nm in size. Abraxane^®^ is currently approved by the FDA for metastatic breast cancer treatment. Polymeric materials have been extensively investigated for biomedical application purposes. As an important branch of DDS, polymeric nanoparticles are the most versatile platform. Anti-cancer drugs can be loaded in different ways: direct conjugation, self-assembly driven by hydrophobic interactions or encapsulation. It is important to note that with the latest advances in polymer science, the polymer skeleton could be endowed with biodegradable properties. In 2007, the approval of Genexol-PM^®^ in Korea marked that first time a polymeric DDS platform was officially put into clinical service.

The translation of DDS-based nanomedicine into clinical practice has offered sufficient improvements to treatment of oncologic diseases. However, it is necessary to note that the major groups of these approved DDS lack active targeting functions and stimuli-responsive controlled release properties. Our understanding of the interaction mechanisms between DDSs and the human body is still insufficient. The safety issue of the designed DDSs still needs full assessment. To address these challenges, unremitting efforts of scientists in different disciplines will accelerate the development of this field.

## 5. Major Challenges and Outlook

In summary, we have reviewed different kind of DDSs according to the categories of their material structure and stimuli-responsiveness functions. Vast amounts of DDSs have been proved to be effective for in vitro drug release. Their unique advantages of high drug loading capacity, stimuli-responsive drug release profiles and biocompatibility have greatly improved the safety and efficacy of conventional small-molecule drugs. However, considering the complexity of the intracorporal environment, translation from bench to bedside remains a huge challenge. Major problems still need to be settled in the following areas:

1. Protein corona effect of nanocarriers. Inside the human body, during the blood transport process, blood proteins have a tendency to cover the surface of DDS through hydrophobic, van der Waals or electrostatic interactions [159]. This phenomenon generally results in enhanced non-specific cellular uptake and has not been intensively studied until recently. The interaction between proteins and nanoparticles is closely related to the physical and chemical properties of the nanomaterial itself. Particle size, charge distribution and other parameters could affect the binding between DDS and proteins. According to the related researches, for silica nanoparticles, polystyrene (PS) nanospheres, and Au nanoparticles, their affinity to protein weakens with decreasing size, but it does not appear to be a universal conclusion; nanoparticle shape also plays a role in protein combination. Serum proteins are mainly negatively charged, therefore, to minimize the serum protein adsorption, DDSs with electronegative surfaces would be better. According to the report of Muller and coworkers, hydrophobic surfaces are prone to be covered by protein substances compared to hydrophilic surfaces. To avoid the protein corona effect, researchers have done systematic work to augment the stealth capability of DDS. PEGylation is a common strategy for prolonging the circulation time and reduce non-specific protein adsorption. Recently, poly(phosphoester)s (PPEs) have been proposed as an effective alternative for PEG.
2. Developing DDS for combination drug therapy. With rapid advancement of cancer treatment studies, single small molecule cancer drugs are no longer the only solution for chemotherapy. Combination drug therapy has been proved to show significantly better outcomes than single drug treatments by suppressing drug resistance and synergistic effects. Recently, interference genes and growth factors have gradually become hot research topics for effective cancer treatment. Together with anti-cancer drugs, this new combination therapy opens up new possibilities for enhanced anticarcinogenic bioactivity [160]. However, current DDSs are limited to the single drug delivery level; few of them have achieved multi-drug co-delivery results. As we have seen from Table 1 and Table 2, the majority of the conceptual DDSs only adopted DOX as model drug. The key problems that hinder successful construction of co-delivery DDSs include: unclear anticancer mechanisms of dual-drugs and precise control of the drug release order.
3. Imperfect tumor targeting effects. Compared with stimuli-responsive drug delivery, site specific drug delivery is an equally important function of an effective DDS. Targeting functions could be built through two mechanisms: passive targeting and active targeting. For passive targeting mode, the key rationale is based on the EPR effect. As the DDS platform becomes diversified, superparamagnetism has been increasingly applied with the introduction of magnetic nanoparticles. Therefore, the location of DDSs inside the human body could be precisely controlled via an external magnetic field. Magnetic orientation has been developed as an effective passive targeting mechanism in recent studies [139,157]. However, passive targeting could not remarkably improve the intracellular uptake of DDS. This inherent limitation has promoted more effective targeting mechanisms. Active targeting mode endows the DDS with “smart judgement” to discriminate the cancer cells. Recent researches revealed that some specific kinds of receptors such as folate and transferrin are overexpressed in cancer cells. Thus bioconjugation of antibodies of these receptors on the surface of nanoscale DDSs can actively deliver DDS into cancer cells. Even though targeted ligands can result in improved cellular uptake, few of them could be clinically applied. This is because the surface properties of the DDSs will be profoundly affected with the conjugation of active targeting ligand and the changes brought by active targeting ligand have yet to be fully understood.
4. Lack of multistimuli-responsive functions. After a DDS enters the human body, we expect it to respond in real time to an efficient stimulus signal and hence, adjust to our demand. However, for single stimulus-responsive DDSs, the drug release process is very likely to be disturbed by extensive interference factors. Due to the heterogeneity of tumor tissues, the pathological characteristics in different kinds of tumors or in different stages of a single tumor are quite diverse. Intelligent DDS, requires an accurate response in a very narrow window. To address this problem, researchers have developed multi-stimuli-responsive mechanisms for building DDSs with high performance. The combination of two or more stimuli-responsive groups are usually parallel or causal. Even more selectivity can be achieved if these responsive groups could be designed to be serial or to work in a Boolean logic pattern. Zink et al. developed an analyte-responsive gate [161]. All stimuli signals were firstly “analyzed” by the smart DDS. The release behavior would only be triggered if two specific stimuli existed simultaneously. In other words, the two specific values are true in this AND gate. If this logic gate could be applied in DDS, stimuli-responsive drug release could be more precisely controlled.
5. Biodegradation problems. After the drug release process, the biodegradation of the DDS becomes an additional problem. As foreign substances can induce an immune response, probably disturbing the anti-tumor drug mechanism. For an ideal, biodegradable DDS, the carrier subject should remain intact. The disassembly process should not take place until the beginning or end of the release behavior. The degraded substances should meet all bio-safety standards and can be easily discharged through metabolism.

As we have sought to show in this review, the successful design of DDSs is a complicated process requiring multi-disciplinary knowledge (pharmacy, biology, chemistry, materials, etc.). For clinical application of DDSs as soon as possible, future research must be based on interdisciplinary cooperation.

## Figures and Tables

**Table 1:** Representative stimuli-responsive drug delivery systems.

Framework	Functional Factor	Payload	Operation Mechanism	Result	Ref.
MSNs	Cyclodextrin based supramolecular nanovalve	*p*-Coumaric Acid	pH-caused dethreading process	Effective pH-triggered release	[84]
MSNs	Pillararene based pseudo[2]rotaxane supramolecular nanovalves	DOX	Acid-induced dethreading process	Proliferation inhibition against MCF-7 cells	[85]
Hollow MSNs	ZnO quantum dot plug gate	DOX	Dissolution of ZnO in acid environment and cleavage of disulfide linker in GSH environment	Anti-proliferative activity against A549 cancer cells	[91]
MSNs	Cyclodextrin based supramolecular nanovalve	DOX & GEM	Acid-induced ketal cleavage and voltage-responsive supramolecular host-guest dedecomplexation	Efficient supression against MCF7 cells	[94]
MSNs	PEG polymer gatekeeper	Dye	Cleavage of disulfide linker in GSH environment	Enhanced delivering effect into cancer cells	[95]
MSNs	PEG based polymer shell, cyclic (Arg-Gly-Asp-d-Phe-Cys) as targeting ligand	Cisplatin/DOX	GSH-induced cleavage of the wrapped polymer shell	Selective drug delivery to KB cells	[96]
MSNs	Dynamic cross-linked supramolecular network of poly(glycidyl methacrylate)s derivative chains	DOX	GSH-induced cleavage of disulfide linker and acid-induced disassembly of the cross-linked polymer network	Good inhibitory effect on A549 cancer cells’ growth	[88]
MSNs	Supramolecular bridge gate of CB[7] and bis-aminated poly(glycerolmethacrylate)s	DOX	Acid and competitive binding caused disassembly of supramolecular bridge gate	Efficient stimuli-responsive drug release both in vitro and in vivo	[98]
MSNs	Lipid bilayer gate and targeting peptide (SP94)	siRNA	Interruption of electronstatic force between MSNs and lipid bilayer caused by acid stimuli	Repressed gene expression at the protein level and cancer cell apoptosis.	[101]
MSNs	Lipid bilayer shell	Calcein	Charge conversion induced cargo release	Successful intracellular delivery of cargo molecules	[103]
Dendrimers	Dendrimer/lipid nanoassemblies	DOX	Lipid layer fusion with the cell membrane caused cargo release	Enhanced cellular uptake of DOX	[116]
Micelles	Amphiphilic poly(acrylamide-co-acrylonitrile)-g-PEG	DOX	Temperature-related solubility change of micelles	temperaturedependent release of DOX	[117]
Vesicles	poly(ethylene oxide)-block-poly(ε-caprolactone)	DOX	Hydrolytic cleavage of caprolactone linker	Prolonging drug retention time, acid stimuli drug release	[118]
Hydrogel	Anionic alginate and cystamine	DOX	Cleavage of disulfide linker in GSH environment	Improved in vitro anticancer efficacy against CAL-72 cells	[105]
MOFs	Carboxylatopillararene based pseudo[2]rotaxanes gatekeeper	DOX	Acid-induced disassembly of pseudorotaxane gatekeeper	pH-sensitive drug release, negligible intrinsic cytotoxicity	[124]
QDs	ZnO@polymer QDs	DOX	Decomposion of ZnO QDs under acid environment	Minimal pre-leakage, controllable drug release within U251 cells, enhanced cell imaging function	[129]
QDs	CQDs and quinoline-chlorambucil units	Chlorambucil	Photo-cleavage of quinoline section under UV irradiation	Photoregulated DOX release within Hela cells	[68]
CNTs	Chitosan coated SWCNTs and FITC fluorescent label	DOX	Weakened π–π stacking function between DOX and SWCNTs under acid environment	Effective intracellular DOX accumulation inside endothelial progenitor cells	[137]
CNTs	Mesoporous coated SWCNTs	DOX	Photothermal heating triggered DOX release under NIR irradiation	Efficient in vivo tumor growth inhibition	[138]
CNTs	FA and iron difunctionalized MWCNT	DOX	Photothermal heating triggered DOX release under NIR irradiation and weakened π–π stacking function under acid environment	Enhanced cancer specificity and drug delivery efficiency	[139]
CNTs	MWCNT-hyaluronic acid conjugate, Alexa-Fluor-647 fluorescent label	DOX	Weakened π–π stacking function in low pH environment	Selective drug accumulation in A549 cells	[140]

MSN, mesoporous silica nanoparticle; DOX, Doxorubicin; GSH, glutathione; GEM, gemcitabine; PEG, polyethylene glycol; CB[7], cucurbituril[7]; MOF, metal-organic framework; QD, quantum dot; CNT, carbon nanotube; SWCNT, single walled carbon nanotube; FITC, fluorescein isothiocyanate; FA, folic acid; MWCNT, multi walled carbon nanotube; NIR, near-infrared.

**Table 2:** Representative examples of DDS therapeutics.

Product	Structure	Active Ingredients	Indication	Stimuli	Status
Doxil^®^	Pegylated liposome	Doxorubicin	Kaposi’s sarcoma, ovarian cancer and breast cancer	-	Approved by FDA
Daunoxome^®^	Liposome	Daunorubicin	Kaposi’s sarcoma	-	Approved by FDA
Caelyx^®^	Liposome	Doxorubicin	Kaposi’s sarcoma, ovarian cancer and breast cancer	-	Approved by European Union
Mepact^®^	Liposome	Muramyl tripeptide phosphatidyl-ethanolamine	Osteosarcoma	-	Approved by European Union
ThermoDox	Liposome	Doxorubicin	Liver cancer and lung cancer	Temperature	Phase III in liver cancer, Phase II in lung cancer
Abraxane^®^	Protein nanoparticle	Paclitaxel	Metastatic breast cancer	-	Approved by FDA
Genexol-PM^®^	Polymeric micelles	Paclitaxel	Breast cancer	-	Approved in Korea
SMANCS^®^	Polymeric conjugate	Neocarzinostatin	Liver cancer	-	Approved in Japan
T-DM1^®^	Antibody conjugate	paclitaxel or docetaxel	Metastatic breast cancer	GSH concentration	Approved by FDA

SMANCS^®^, poly(styrene-co-maleic acid)-conjugated neocarzinostatin; T-DM1, trastuzumab emtansine-mertansine.
